# High Throughput Screening of Transcription Factor LysG for Constructing a Better Lysine Biosensor

**DOI:** 10.3390/bios14100455

**Published:** 2024-09-25

**Authors:** Qinggang Li, Haojie Ren, Zhenjiang Liao, Shuchang Xia, Xue Sun

**Affiliations:** 1 Key Laboratory of Industrial Fermentation Microbiology of the Ministry of Education, Tianjin Key Laboratory of Industrial Microbiology, College of Biotechnology, Tianjin University of Science and Technology, National Engineering Laboratory for Industrial Enzymes, Tianjin 300457, China; 2 Haihe Laboratory of Synthetic Biology, Tianjin 300308, China

**Keywords:** transcription factors, biosensor, LysG, high throughput screening

## Abstract

The biosensors based on transcription factors (TFs) are widely used in high throughput screening of metabolic overproducers. The unsatisfactory performances (narrow detection and dynamic ranges) of biosensors limit their practical application and need more improvement. In this study, using the TF LysG (sensing lysine) as an example, a biosensor optimization method was constructed by growth-coupled screening of TF random mutant libraries. The better the performance of the biosensor, the faster the strain grows under screening pressure. A LysG^E15D, A54D, and I164V^-based biosensors were obtained, which were about 2-fold of the control in the detection and dynamic ranges. A lysine high-producer was screened effectively using the optimized biosensor with the production at 1.51 ± 0.30 g/L in flasks (2.22-fold of the original strain). This study provided a promising strategy for optimizing TF-based biosensors and was of high potential to be applied in the lysine high-producers screening process.

## 1. Introduction

High throughput screening (HTS) based on biosensors is a useful technology for continuously increasing the production of metabolites by microorganisms [1,2,3,4]. Transcription factors (TFs) serve as essential elements and are commonly used in biosensor designs [5,6]. TFs could discriminate the concentrations of intracellular metabolites (input signals) and translate them into distinguishable forms (output signals) with high efficiency and precision [3,7].

The detection and dynamic ranges of biosensors are defined by the concentration range of the metabolite being detected as the input signal (horizontal input) and the maximum fold change in the reporter output signal (vertical output), respectively [5,8,9]. For biosensors based on native TFs, the detection and the dynamic ranges are usually limited by various factors, including the stringent regulatory mechanisms of TFs [10,11]. These limitations could be relieved through the optimization of TFs, which includes both rational and irrational design strategies [12,13]. While the rational design strategy often requires comprehensive structural and mechanistic information, the irrational design strategy has been the prevalent optimization method because of its universality [5]. However, the irrational design process often results in a large number of random mutants. Consequently, it is necessary to develop an effective screening method to identify superior mutants from extensive mutation libraries.

In addition to TFs, DNA aptamers, and riboswitches also serve as key components in biosensors. The systematic evolution of ligands by exponential enrichment (SELEX) is a widely used technique in the selection of DNA aptamers libraries [14], and an effective method for altering the threshold of riboswitch has also been established based on growth-coupled screening [15]. However, there is a shortage of reported methods for screening superior TF mutants from mutation libraries.

The TF LysG is a member of the extensive LysR-type family and could respond to lysine, a crucial amino acid with significant applications in the animal feed, food, and medicine industries [16]. In the presence of lysine, LysG binds to the promoter P*_lysE_* and activates the expression of the downstream gene *lysE*, which is responsible for the efflux of lysine [17]. LysG has been developed for constructing lysine biosensors in *Corynebacterium glutamicum* (*C. glutamicum*) [18].

In this study, a screening method to identify superior TF from mutation libraries was developed, using LysG as an example. First, a lysine biosensor was constructed in *Escherichia coli* (*E. coli*) based on LysG from *C. glutamicum*. The *tetA* gene was used as the report gene, which encodes the tetracycline efflux ABC transporter. In the presence of 5 g/L lysine, the biosensor strain exhibited a 30-fold increase in tetracycline resistance compared with the control without lysine. The detection and dynamic ranges of the biosensor were subsequently optimized through the screening of LysG mutant libraries. The better the LysG mutant, the higher the expression level of TetA, and the better the strain growth under high tetracycline stress. Identifying better LysG mutants by observing strain growth on agar plates under tetracycline pressure is convenient. Moreover, the performance of the biosensor was significantly optimized, with both the detection and dynamic ranges being approximately 2-fold that of the control. Screening used the optimized biosensor; a lysine mutant producer was obtained. The lysine production was 1.51 ± 0.30 g/L in flasks (2.22-fold of the original strain). This study provided a promising screening strategy for TF-based biosensors’ optimization and was beneficial for the screening process of lysine overproducers.

## 2. Materials and Methods

### 2.1. Enzymes and Chemicals

The DNA polymerase used for fragment amplification in PCR was sourced from Transgene (Beijing, China). The Taq polymerase used for PCR verification was acquired from Cwbio (Beijing, China). The DNA Purification Kit was supplied by TIANGEN (Beijing, China). The CloneExpress II/MultiS One Step Cloning kit was provided by Vazyme (Nanjing, China). The *E. coli* strain DH5α was also procured from Transgene. Oligonucleotides were synthesized by GENEWIZ (Beijing, China). Lysine and antibiotics were obtained from Solarbio (Beijing, China).

### 2.2. Cultivation Media

The Luria–Bertani (LB) medium contains 5 g/L yeast extract, 10 g/L tryptone, and 10 g/L NaCl. The M9 medium contains 10 g/L yeast extract, 10 g/L tryptone, 3 g/L KH_2_PO_4_, 0.01 g/L MnSO_4_·H_2_O, 0.01 g/L FeSO_4_·7H_2_O, 1 g/L MgSO_4_·7H_2_O, 10 g/L (NH_4_)_2_SO_4_, 40 g/L MOPS, and 20 g/L glucose. Each medium had a pH level of 7.0. The agar plates were prepared by incorporating 2% agar into the liquid medium. Depending on the strains’ resistance, ampicillin (100 mg/L) and/or kanamycin (50 mg/L) were included in the media. For the constructions of plasmid and strains, cultures were grown in test tubes with shaking at 220 rpm and either 37 °C or 30 °C (specifically for CRISPR/Cas9-assisted gene edition) in LB medium. The growth test of strains was conducted by cultivating the strains on M9 medium agar plates with tetracycline. When conducting fermentation, strains were cultivated in 24-deep-well plates (shaking at 800 rpm) or shake flasks (shaking at 220 rpm) at 37 °C in an M9 medium without tetracycline.

### 2.3. Plasmid Construction

The main bacterial strains, plasmids, and primers used in this study are listed in Supporting Information (SI, Table S1). The *E. coli* strain DH5α was used for the plasmid construction and is cultivated routinely using LB medium. For overexpressing gene *lysG*, promoter P*_lysE_*, and gene *tetA*, the plasmid pSB4K5-*lysG*-P*_lys__E_*-*tetA* was constructed on the basis of a former plasmid pSB4K5 [19]. The plasmid backbone pSB4K5, the *lysG* gene, promoter P*_lysE_*, and the *tetA* gene were amplified using primer pairs psb-F/R, *lysG*-F/R, and *tetA*-F/R from plasmid pSB4K5, *C. glutamicum* ATCC13032 genomic DNA and plasmid pDM1, respectively. The three DNA fragments were ligated using the CloneExpress MultiS One Step Cloning Kit to generate plasmid pSB4K5-*lysG*-P*_lys__E_*-*tetA*.

For overexpressing wild-type gene *lysC*, the plasmid pTrc99a-*lysC* was constructed on the basis of plasmid pTrc99a. The plasmid backbone pTrc99a and the *lysC* gene were amplified using primer pairs 99a-F/R and lysC-F1/R1 from plasmid pTrc99a and *E. coli* MG1655 genomic DNA. The two DNA fragments were ligated using the CloneExpress MultiS One Step Cloning Kit to generate plasmid pTrc99a-*lysC*. The mutations T352I and S369F of gene *lysC* were conducted using the primers lysC-F2/R2 and lysC-F3/R3, obtaining plasmid pTrc99a-lysC* [20].

For overexpressing wild-type *dapA*, the plasmid pTrc99a-*dapA* was constructed on the basis of plasmid pTrc99a [19]. The plasmid backbone pTrc99a and the *dapA* gene were amplified using primer pairs 99a1-F/R and dapA-F1/R1 from plasmid pTrc99a and *E. coli* MG1655 genomic DNA. The two DNA fragments were ligated using the CloneExpress MultiS One Step Cloning Kit to generate plasmid pTrc99a-*dapA*. The mutations H118Y and A81V of gene *dapA* were conducted using the primers dapA-F2/R2 and dapA-F3/R3, obtaining plasmid pTrc99a-dapA* [21].

For overexpressing gene *ddh*, the plasmid pTrc99a-*ddh* was constructed on the basis of plasmid pTrc99a. The plasmid backbone pTrc99a and the *ddh* gene were amplified using primer pairs 99a2-F/R and *ddh*-F/R from plasmid pTrc99a and *C. glutamicum* A21799 genomic DNA. The two DNA fragments were ligated using the CloneExpress MultiS One Step Cloning Kit to generate plasmid pTrc99a-*ddh*.

### 2.4. Strain Construction

The CRISPR/Cas9-assisted gRNA-free one-step (CAGO) genome editing technique (the gRNA needed for editing is universal) was utilized for genome gene deletion and overexpression [22]. In this technique, a homologous recombinant fragment is needed with flanking homology arms and a chloramphenicol marker gene *cat*. And for gene overexpression, the gene should be added downstream of the *cat* marker gene. Using the *E. coli* MG1655 genome (GenBank accession No. NC_000913) and lab-stored *cat* gene as templates and primers detailed in Supplementary Information (SI, Table S1), the homologous recombinant fragments were generated by PCR. Subsequent recombination was performed following the established steps [22].

For strain P1, the *lysC*^T352I, S369F^ mutant gene was integrated into the genome via homologous recombination. The fragment for recombination was constructed by ligation of the fragments amplified with primers UP-C-F/R, lysC-cat-F/R, and lysC-F/R, using the MG1655 genome, lab-stored *cat* gene, and pTrc99a-lysC* as templates separately.

Strain P2, derived from P1, had the *dapA*^H118Y, A81V^ mutant gene integrated into the genome. Similarly, the fragment for recombination was constructed by ligation of the fragments amplified with primers UP-A-F/R, dapA-cat-F/R, and dapA-F/R, using the MG1655 genome, lab-stored *cat* gene, and pTrc99a-dapA* as templates separately.

Plasmid pTrc99a-*ddh* was introduced into strains P1 and P2 to obtain strains P3 and P4, respectively. Control strains P5 was obtained through transforming plasmid pTrc99a to MG1655.

For strains equipped with the LysG-based biosensor (referred to as biosensor strains), plasmid pSB4K5-*lysG*-P*_lysE_*-*tetA* was introduced into MG1655 to obtain strain S1. For the construction of strains S3, S4, and S5, the plasmid pSB4K5-*lysG*^M^-P*_lys__E_-tetA* was transformed into strains P1, P4, and P5, respectively.

### 2.5. Screening of LysG Mutant from Random Mutation Library

First, the LysG random mutation library was constructed using the error-prone PCR. The gene *lysG* was amplified using plasmid pSB4K5-*lysG-*P*_lys__E_-tetA* as the template with primers EG-F/R. Moreover, the primers EGV-F/R were used to amplify the linear vectors without *lysG* using plasmid pSB4K5-*lysG-*P*_lys__E_-tetA* as the template. The fragments containing different lysG mutants were linked to linear vectors using the CloneExpress MultiS One-Step Cloning Kit. The plasmids with different *lysG* mutants were transformed into strain MG1655, obtaining the LysG random mutation library (about 10^5^ mutants). After cultivating to an OD_600_ of 1.4 in LB medium, 1 mL culture was diluted 10^5^ times using an M9 medium. Then, 0.1 mL culture was spread over M9 agar plates containing different concentrations of tetracycline and lysine. After cultivation at 37 °C for 60 h, the growth of these mutants on plates was compared.

### 2.6. Screening Lysine Overproducers from Mutant Libraries

First, strain P4 was used for random mutagenesis through Atmospheric and Room Temperature Plasma (ARTP) irradiation. The ARTP irradiation was performed as described former (with the power at 125 W, flux 10 SLM, and 60 s) [7]. Five samples after ARTP irradiation were individually inoculated to an OD_600_ of 0.6 and prepared as competent cells. The biosensor plasmid pSB4K5-*lysG*^M^-P*_lys__E_-tetA* was transformed into mutants subsequently. Strain S4 was used as the control strain. After cultivation at 37 °C for 12 h, different mutants from each sample were spread over M9 agar plates with tetracycline. After cultivating for 60 h on the plates, the bigger colonies were randomly selected. The growth and lysine concentrations of these mutants were tested in the M9 medium without tetracycline.

### 2.7. Cell Growth and Lysine Fermentation Analysis

To evaluate the impact of different lysine or tetracycline concentrations on cell growth, the lysine or tetracycline was added to the medium to achieve the desired final concentrations individually. Strains were initially grown in LB medium overnight and resuspended in a 10-fold volume of medium after centrifugation. After an additional 12 h incubation, the cultures were diluted by 10^6^ folds using an M9 medium. The diluent (100 μL) was spread onto M9 plates cultivating at 37 °C for 60 h. Colony images were obtained using the Tanon 1600 Gel Image System (Shanghai, China) and the sizes were compared according to the colony pixels through the software provided with the instrument [3].

Lysine productions were measured by high-performance liquid chromatography (HPLC) using an evaporative-light-scattering detector from Agilent (Santa Clara, CA, USA) and a carbohydrate column (ES 5 μm 250 mm × 4.6 mm), following the established method [23]. The concentration of lysine was calculated through a calibration curve obtained with the standard lysine solution.

## 3. Results and Discussion

### 3.1. Design and Proof-of-Concept of the Biosensor Based on LysG

*E. coli* strain is widely used as the host strain for lysine production with simple cultivation conditions and high production efficiency. The lysine production could be increased by both rational or irrational design in *E. coli*. HTS based on biosensors is an effective method for screening strains with increased lysine production [24]. The TF LysG is sensitive to lysine levels, could bind to the P*_lysE_* promoter, and thereby activate the expression of the *lysE* gene, which encodes a lysine efflux transporter [17]. The biosensor based on LysG has been constructed in *C. glutamicum* [18,25]. However, with increases in lysine production, the detection and dynamic ranges of existing biosensors were limited in practical applications. In this study, a novel biosensor was constructed in *E. coli*, based on LysG from *C. glutamicum* and the report gene *tetA*. The gene *tetA* encodes the tetracycline efflux ABC transporter, and its expression level is indicative of strain growth under tetracycline stress [26]. In theory, the lysine production and strain growth could be coupled through the biosensor conveniently. The growth of various strains could be conveniently and efficiently distinguished on agar plates, offering a practical method for strain screening [3,27].

**Figure 1:**
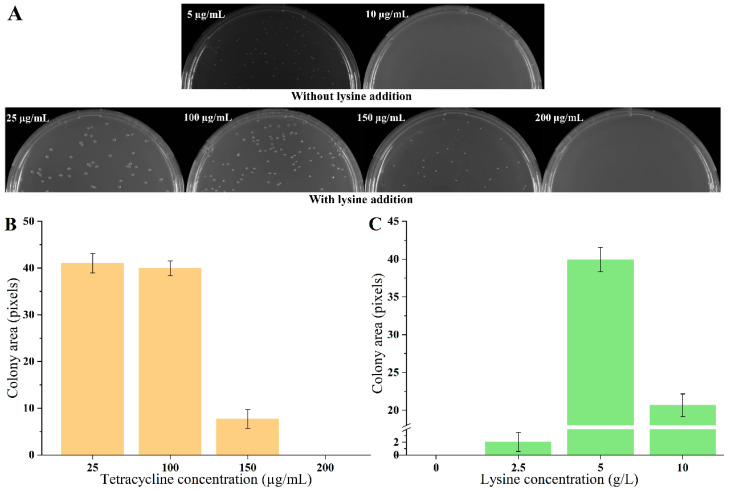
Growth of biosensor strains on agar plates. (**A**) The growth of strain S1 on agar plates with different concentrations of tetracycline and without/with 5 g/L lysine. (**B**) The strain growth with 25, 100, 150, 200 µg/mL tetracycline and 5 g/L lysine. (**C**) The strain growth with 0, 2.5, 5, and 10 g/L lysine and 100 µg/mL tetracycline. Data are shown as the mean and standard deviation of independent triplicates.

The biosensor plasmid pSB4K5-*lysG-*P*_lys__E_-tetA* was constructed and transformed into strain *E. coli* MG1655, obtaining biosensor strain S1. The growth of strains was detected on plates containing different concentrations of tetracycline (5, 10, 15, 20, and 25 µg/mL, respectively) in the absence of lysine. The growth of strains was detected after 60 h cultivation, using the detection method described in 2.7. The colony size of strain S1 was 5.35 under 5 µg/mL tetracycline. There was no colony on plates with tetracycline exceeding 10 µg/mL. Then, 5 g/L lysine was added to the medium. With the addition of lysine, the growth of all strains improved, with no significant differences observed on plates containing 10, 15, 20, and 25 µg/mL tetracycline. To elucidate the impact of varying lysine concentrations on strain growth clearly, tetracycline concentrations were increased to 100, 150, 200, 250, and 300 µg/mL. Notably, in the presence of 5 g/L lysine, no strain growth was detected on plates with tetracycline concentrations exceeding 150 µg/mL (Figure 1A,B). Employing the tetracycline-resistant level to define the dynamic range, it was evident that the addition of 5 g/L lysine significantly induced the expression of TetA, demonstrating that the dynamic range of biosensor was 30-fold (from 5 µg/mL to 150 µg/mL). The detection range of the biosensor was further evaluated with different concentrations of lysine addition (0, 2.5, 5, and 10 g/L) under the 100 µg/mL tetracycline condition. There were growth enhancements continuous for strain S1 with increasing lysine concentrations from 0 to 5 g/L (Figure 1C). However, the addition of 10 g/L lysine led to a growth inhibition for strain S1. These results indicated that the maximal lysine detection concertation of biosensors was 5 g/L.

A lysine biosensor was constructed in *E. coli* based on LysG from *C. glutamicum*. The growth level of the biosensor strain was coupled with the concentration of lysine. The biosensor strain could grow in the presence of 5 µg/mL tetracycline even without lysine addition, indicating a basal level of TetA expression. The basal expression of report gene *tetA* might be caused by leakage expression of promoter P*_lysE_*, which might be similar to P*_trc_* [28,29]. In the presence of 5 g/L lysine, the biosensor strain was able to grow under a tetracycline concentration of 150 µg/mL. There was a dynamic range of 30-fold, signifying a substantial activation effect of lysine on the biosensor strains. In addition, the fold induction was notably enhanced compared with the previously developed LysG-based biosensor (4.21-fold and 8.30-fold in *C. glutamicum*) [12,18]. The LysG used here in *E. coli* originated from *C. glutamicum*. LysG activated the expression of LysE, which is responsible for lysine efflux [17]. If a LysG-based biosensor is constructed in *C. glutamicum*, the overexpression of LysG on plasmid might interfere with lysine metabolism. However, when overexpressing LysG from *C. glutamicum* in *E. coli*, LysG played the role of sensing lysine and activating TetA. The lysine metabolic pathway might not be interfered with, which might result in a larger difference in the output signal [30]. Although biosensors have broad prospects in various applications, the universality of biosensors among different hosts is a significant challenge [2]. In this study, the TF LysG from *C. glutamicum* was engineered to function as a biosensor in heterologous host *E. coli* successfully. In addition, it is essential to develop stable and universally applicable sensing elements that can function across different hosts.

### 3.2. Optimization of the Biosensor through Screening LysG Mutant

**Figure 2:**
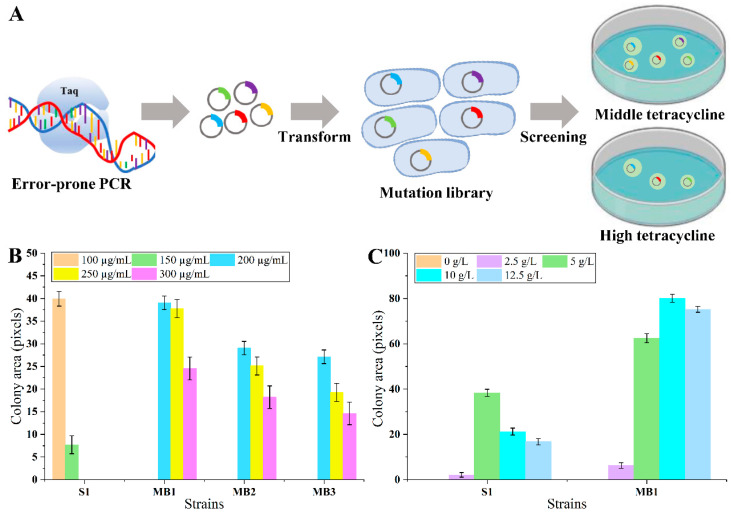
The scheme of LysG optimization process and the growth of biosensor strains on agar plates. (**A**) The scheme of LysG optimization process. (**B**) The growth of LysG-mutant-based biosensor strains and control strain S1 under the conditions of 100, 150, 200, 250, and 300 µg/mL tetracycline and 5 g/L lysine. (**C**) The growth of strain MB1 and control strain S1 under the condition of 0, 2.5, 5, 10, and 12.5 g/L lysine and 100 µg/mL tetracycline; Strain MB1 was the biosensor strain with mutant LysG, and strain S1 was the biosensor strain with wild-type LysG. Data are shown as the mean and standard deviation of independent triplicates.

The current maximal detectable lysine concertation of the biosensor might not be adequate for high producers [16]. To distinguish the strain growth more obviously during high-throughput screening, it was essential to optimize the biosensor for broader detection and dynamic ranges, which could be more practical for applications. The optimization has been realized through LysG rational or semi-rational design [12,31]. However, the structure and mechanism of LysG were intricate, which means that rational design might not be highly accurate and require numerous attempts. Meanwhile, semi-rational design might lead to time and labor costs because of the need for individual evaluation. An efficient method was urgently needed to obtain LysG mutant with superior performance. If using the tetracycline tolerance levels of biosensor strains as the dynamic range (with sufficient lysine to induce the maximum expression of TetA), theoretically, the larger the dynamic range, the higher the expression level of TetA and the better the strain growth under certain tetracycline stress (As shown in Figure 2A). In detail, in the presence of different tetracycline concentrations, there should be different growth of strains with different LysG mutants. At low tetracycline concentrations, there should be no growth difference. At middling tetracycline concentrations, there should be different growth between mutants (the larger the dynamic range, the better the strain growth). When the tetracycline concentration surpasses the threshold at which the strain with the original LysG-based biosensor ceases to grow, only the strains equipped with a LysG mutant-based biosensor that enlarges dynamic range could grow. The dynamic range could be improved by detecting strain growth. There is great research potential regarding this screening method because of the diversity of TF types (more than 230 TFs in *E. coli*) [18].

Random mutation of LysG was performed using error-prone PCR, obtaining a LysG-mutant-based biosensor library. The library was transformed into strain MG1655. To obtain LysG mutants with a larger dynamic range, these mutants were cultivated on plates containing 5 g/L lysine and 200 µg/mL tetracycline, a condition that is inhibitory to the growth of the original biosensor strain. After 60 h cultivation, the colony sizes were compared. Three colonies exhibiting larger sizes (named strain MB1, MB2, and MB3) were selected for further analysis. The growth was detected on plates containing varying concentrations of tetracycline (200, 250, 300, 400, and 500 µg/mL) and 5 g/L lysine. As shown in Figure 2B, all three strains demonstrated improved growth than the original strain, and strain MB1 grew best at 300 µg/mL tetracycline. However, when the tetracycline concentration exceeded 400 µg/mL, the growth of all three strains was inhibited. It implied that the dynamic range of the biosensor was successfully expanded 2-fold compared with the control (from 150 to 300 µg/mL tetracycline) in the presence of 5 g/L lysine.

The change in dynamic range might lead to negative interfere to the detection range during prior optimization processes [12]. To verify whether there was an impact on the detection range, the growth of strain MB1 was detected further under conditions with varying concentrations of lysine. Strain MB1 and the control strain S1 were cultivated on plates containing 100 µg/mL tetracycline and lysine concentrations of 0, 2.5, 5, 10, and 12.5 g/L, respectively. As shown in Figure 2C, strain MB1 grew significantly better with 10 g/L lysine compared with the control strain S1. When added 12.5 g/L lysine, the colony area of strain MB1 became slightly smaller compared with the colony area with the addition of 10 g/L lysine, indicating that the detection range of the biosensor in MB1 was 0–10 g/L, and the smaller colony might be caused by the pressure of high lysine concentration. Consequently, for the biosensor strain MB1, the lysine detection range was extended from 5 g/L to 10 g/L.

The improvement of biosensors’ detection and dynamic ranges is challenging [5]. Desired mutants are often exceedingly rare within libraries. It is convenient to distinguish strains with distinct growth differences through screening on agar plates [1]. Utilizing the LysG mutation and screening strategy developed in this study, three mutants were identified from a large library containing approximately 10^5^ mutants. Both the detection and dynamic ranges of the superior mutant strain MB1 were about 2-fold of the control. Compared with the conventional rational or semi-rational methods [12,31], the approach in this study proved to be more convenient and efficient. This strategy has not been reported previously and may be applicable for the optimization of numerous biosensors based on TFs.

**Figure 3:**
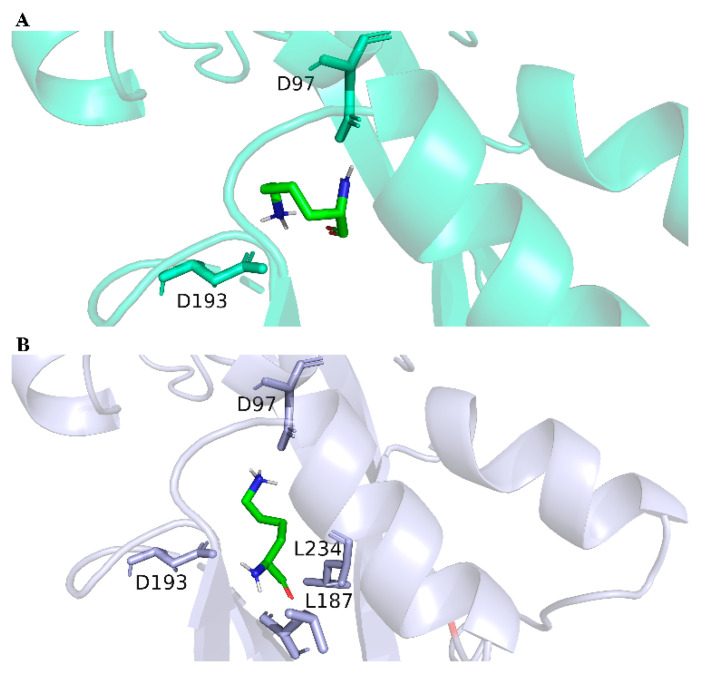
The structures of LysG wild-type and LysG^M^ after binding with lysine. (**A**) The structure of LysG wild-type after binding with lysine. (**B**) The structure of LysG^M^ after binding with lysine.

The LysG in strain MB1 was sequenced, and there were three mutation sites (E15D, A54D, and I164V) in the LysG mutant. This mutant was designated as LysG^M^. In general, the mechanism changes in TFs were usually caused by structural alterations [32]. In this study, the change in the binding mechanism of LysG with lysine was not the main focus. The point of this study was to provide an HTS method for obtaining TFs with better performance, as well as a method for constructing more efficient lysine biosensors. We had only conducted molecular dynamics simulations using the software GROMACS 2021.2, which is widely recognized by researchers [33]. The impacts of these mutations on the structure and binding affinity were elucidated through a series of simulations. As shown in Figure 3 and Supporting Information (SI, Tables S2 and S3), the binding energy between LysG^M^ and lysine was reduced compared with the wild-type LysG. It indicated a more stable binding interaction between LysG^M^ and lysine. Moreover, the simulation results indicated that LysG^M^ forms a greater number of polar contacts with lysine than the wild-type. These findings suggested that the binding of LysG^M^ to lysine was facilitated, potentially enhancing the detection capacity for lysine.

### 3.3. Endogenous Lysine Overproduction Enhanced Growth of the Biosensor Strains

**Figure 4:**
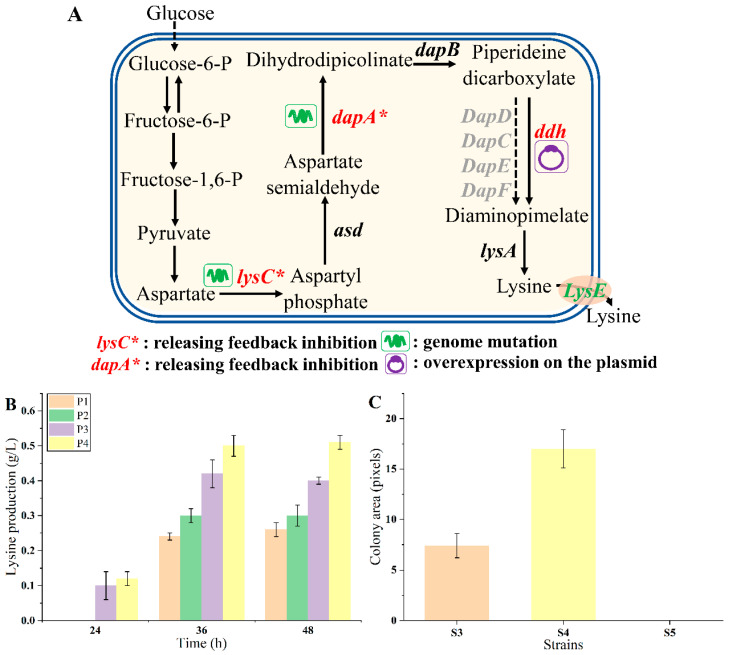
The endogenous lysine overproduction. (**A**) The rational design of lysine pathway. (**B**) The endogenous lysine production of strains P1, P2, P3, and P4 after 24, 36, and 48 h fermentation, respectively. (**C**) The growth of strains with different lysine production on plates. Strain S4 was a higher lysine producer, and strain S3 was a lower lysine producer. Data are shown as the mean and standard deviation of independent triplicates.

To verify whether endogenous lysine biosynthesis could enhance the growth of biosensor strains, strain MG1655 was chosen as the chassis for constructing lysine overproducers due to its convenient genetic manipulation and cultivation. As shown in Figure 4A, the aspartate kinase (LysC) and dihydrodipyridyl dicarboxylic acid synthase (DapA) are feedback inhibited by lysine, as previously reported [34,35,36]. To release the inhibition, the *lysC*^T352I, S369F^ mutant (designated as lysC*) was genetically integrated into the genome of strain MG1655, obtaining strain P1. The *dapA*^H118Y, A81V^ mutant (named dapA*) was further introduced into the P1 genome, obtaining strain P2. Meanwhile, the diaminohexate dehydrogenase gene (*ddh*) from *C. glutamicum* was introduced via the plasmid pTrc99a-*ddh* into strains P1 and P2, obtaining strains P3 and P4, respectively [37,38]. A control strain P5 was constructed by transferring the empty vector pTrc99a into strain MG1655. The lysine production of these strains was tested in 24-deep-well plates using M9 medium. As shown in Figure 4B, the lysine production of strains P1, P2, P3, and P4 were 0.26 ± 0.02, 0.30 ± 0.03, 0.40 ± 0.01, and 0.51 ± 0.02 g/L at 48 h, respectively. The metabolic engineering strategies employed were effective, enhancing lysine production.

The LysG^M^-based biosensor was transformed into strain P1 (characterized by lower lysine production), P4 (characterized by higher lysine production), and P5 (lacking lysine production), obtaining biosensor strains S3, S4, and S5, respectively. The response of the biosensor to lysine was examined by detecting the growth of the biosensor strains on plates with 10 µg/mL tetracycline. Under this condition, the control strain S5 was unable to grow. The colony size of S4 was about 2.30-fold compared with that of S3 (Figure 4C). These results suggested that the endogenous lysine production level and the growth of biosensor strains were positively correlated. The refined LysG biosensor effectively distinguished strains with discernible growth differences on plates, which was convenient [3,27,39]. Consequently, it should be feasible to select the desired lysine overproducers on plates according to the colony size directly using this biosensor.

### 3.4. High Throughput Screening of Lysine Overproducers from Random Mutation Libraries

**Figure 5:**
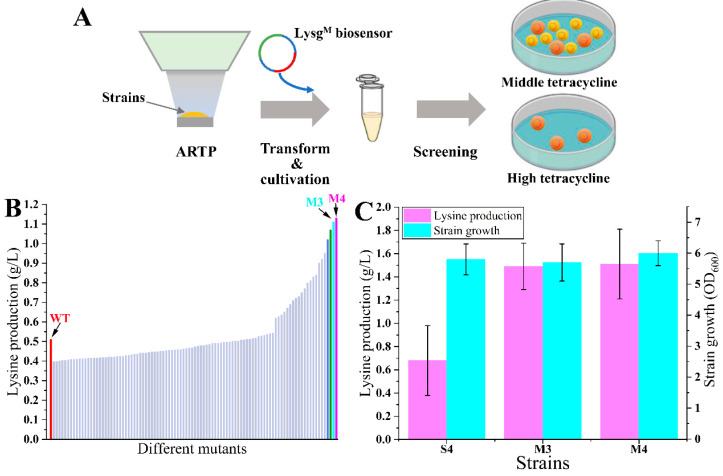
Screening of lysine overproducers. (**A**) The scheme of lysine overproducers screening process. (**B**) The lysine production of different mutant strains and control strain S4 (shown as WT) in 24-deep-well plates. (**C**) The shake-flask fermentation of mutant-producing strains M3, M4, and control strain S4. Data in B are shown as the mean and standard deviation of independent triplicates.

The selection of desired lysine overproducers from the random mutant libraries was conducted using the optimized biosensor (Figure 5A). The lysine producer P4 was selected as the original strain. Five replicate cultures of strain P4 were subjected to ARTP irradiation to generate random mutant libraries. The biosensor plasmid pSB4K5-*lysG*^M^-P*_lys__E_-tetA* was transformed into each library (about 10^5^ living cells each) after ARTP irradiation to avoid potential mutations in the biosensor. Then, the screening was performed directly on M9 medium plates with 15 µg/mL tetracycline. After cultivating at 37 °C for 60 h, the control strain S4 could not grow on plates. A total of 100 large colonies (designated M1 to M100) were randomly selected for further evaluation through fermentation in 24-deep-well plates. There were 32 mutants with higher lysine production compared with strain S4 after 48 h fermentation. Most of these mutants showed slight increases, while two mutants, M3 and M4, performed excellently with the lysine production at 1.11 and 1.13 g/L, respectively (Figure 5B). Shake-flask fermentation of mutants M3 and M4 was conducted in the M9 medium without tetracycline addition. The results revealed significant increases in lysine production, which were 2.19-fold (1.49 ± 0.20 g/L) and 2.22-fold (1.51 ± 0.30 g/L) of the original strain (0.68 ± 0.30 g/L), respectively (Figure 5C). However, the purpose of this section was mainly to validate the feasibility of the LysG^M^-based biosensor. Given that the original strain was the MG1655 wild-type, the lysine production of currently obtained mutants was still low. It was not very meaningful to analyze the detailed mechanism of these mutants. In the next work, this screening method could be further utilized for the screening of lysine high-producers. It could be more significant to elucidate the mechanisms that contribute to the enhanced lysine production capabilities of these high-producing mutants.

Through ARTP irradiation, a substantial number of diverse mutants were generated, including both beneficial and unfavorable variants [4]. Consequently, it was necessary to employ a rapid and efficient method for screening the desired mutant from the mutant libraries [3]. Using the LysG^M^-based biosensor, the mutants obtained with better growth on plates certainly increased the lysine production. It indicated a high level of efficiency in the screening process for lysine overproducers from the mutant libraries. A mutant lysine producer was successfully isolated. The lysine production of this mutant strain was 1.51 ± 0.30 g/L, which was 2.22-fold of the original strain. Here, we employed strains with relatively lower lysine production as an example, proving the efficacy of the engineered biosensor in screening. Since the construction of high-producing strains is still underway, we will validate the method with more examples in the next work. Nevertheless, this research should provide a beneficial method for other researchers to screen for lysine high-producing strains.

## 4. Conclusions

A lysine biosensor was constructed in *E. coli*, utilizing the LysG derived from *C. glutamicum*. The growth level of the biosensor strain was coupled with the lysine concentration. Further, the detection and dynamic ranges of the biosensor were optimized through LysG mutation and screening strategy. The performance of the refined biosensor exhibited a significant improvement, with both the detection and dynamic ranges being approximately 2-fold of the unmodified biosensor. Utilizing this optimized biosensor, a mutant strain with improved lysine production was obtained. The lysine production of the mutant strain was 1.51 ± 0.30 g/L, which was 2.22-fold of the original strain. This investigation was beneficial for the screening of lysine high-producers and offered a valuable strategy for optimizing TF-based biosensors.

## Data Availability

Data presented in this study are available on request from the corresponding author.

## Supplementary Materials

The following supporting information can be downloaded at: https://www.mdpi.com/article/10.3390/bios14100455/s1, Table S1: Main strains, plasmids, and primers used in this study; Table S2: The binding energy of LysG wild-type and lysine; Table S3: The binding energy of LysG^M^ and lysine.
