# Supplementary material for: High Throughput Screening of Transcription Factor LysG for Constructing a Better Lysine Biosensor
Source: Biosensors (Basel). 2024 Sep 25;14(10):455. doi: 10.3390/bios14100455 (PMC11506072; doi:10.3390/bios14100455)
Supplement: Supplementary file 1 [file biosensors-14-00455-s001.zip › biosensors-3180504-supplementary.pdf]

biosensors

MDPI

Supplementary material

Table S1. Main strains, plasmids, and primers used in this study.

Strain/plasmid/primer	Description	Source
**Strain**		
MG1655	A substrain of *E. coli* K-12 strain, with a relevant GenBank accession No: NC_000913	Lab stock
E. coli DH5α	Gene cloning	Lab stock
C. glutamicum A21799	Provide gene *ddh*	Lab stock
C. glutamicum A13032	Provide gene *lysG*	Lab stock
S1	MG1655(pSB4K-*lysG*-P_{lysE}-*tetA*)	This study
MB1	MG1655(pSB4K5-*lysG**-P_{lysE}-*tetA*)	This study
P1	MG1655 with *lysC*$^{T352I,\ S369F}$	This study
P2	P1 with *dapA*$^{H118Y,\ A81V}$	This study
P3	P1 (pTrc99a-*ddh*)	This study
P4	P2 (pTrc99a-*ddh*)	This study
P5	MG1655 with pTrc99a	This study
S3	P1 (pSB4K5-*lysG**-P_{lysE}-*tetA)*	This study
S4	P4 (pSB4K5-*lysG**-P_{lysE}-*tetA)*	This study
S5	P5 (pSB4K5-*lysG**-P_{lysE}-*tetA)*	This study
**Plasmid**		
pCAGO	Ap^{R}, containing Cas9 and gRNA	Lab stock
pTrc99A	Ap^{R}, GenBank accession no.: U13872	Lab stock
pDM1	Provides tetracycline resistance gene *tetA*	Lab stock
pTrc99a-*lysC*	pTrc99A derivative, using promoter P_{trc} to overexpress *lysC*	This study
pTrc99a-*lysC**	pTrc99A derivative, using promoter P_{trc} to overexpress *lysC*$^{T352I,\ S369F}$	This study
pTrc99a-*dapA*	pTrc99A derivative, using promoter P_{trc} to overexpress *dapA*	This study
pTrc99a-*dapA**	pTrc99A derivative, using promoter P_{trc} to overexpress *dapA*^{H118Y,A81V}	This study
pTrc99a-*ddh*	pTrc99A derivative, using promoter P_{trc} to overexpress *ddh*	This study
pSB4K5	Km^{R}, pSB4K5-I52002 (GenBank accession no.: EU496099) backbone	Lab stock
pSB4K5-*lysG-tetA*	pSB4K5 derivative, using promoter P_{trc} to overexpress *lysG* , promoter P_{lysE} and gene *tetA*	This study
pSB4K5-*lysG**-*tetA*	pSB4K5 derivative, using promoter P_{trc} to overexpress *lysG**, promoter P_{lysE} and gene *tetA*	This study
**Primer**		
psb-F	cgggccacctcgacctgaaacccttctcacctcggc	
psb-R	tcgagggattgcggccttaggcctggggtgcctaatga	
lysG-F	ctaaggccgcaatccctcga	
lysG-R	atgtatatctccttcttaaagtcatctagg	
tetA-F	agatgactttaagaaggagatatacatatgaaatctaacaatgcgct	
tetA-R	tcaggtcgaggtggcccg	
99a-F	atagtaatttgtttgagtaatcgctcaaggcgcactcc	
99a-R	tggagacaacaatttcagacatcacgaattatgcagtgatttacgacc	
lysC-F1	atgtctgaaattgttgtctccaa	
lysC-R1	ttactcaaacaaattactatgcagttt	
lysC-F2	gtggcattaatccttgataccacc	
lysC-R2	ggtggtatcaaggattaatgccac	
lysC-F3	tgacgcaatttctgctgatg	
lysC-R3	catcagcagaaattgcgtca	
99a1-F	taagcatgccggtttgctgtaatcgctcaaggcgcactcc	
99a1-R	gacaatacttcccgtgaacatcacgaattatgcagtgatttacgacc	

dapA-F1	atgttcacgggaagtattgtc
dapA-R1	ttacagcaaaccggcatg
dapA-F2	ttgtatcagtatttcaaagccatcg
dapA-R2	cgatggctttgaaatactgatacaa
dapA-F3	cgctaacgttactgcgacc
dapA-R3	ggtcgcagtaacgttagcg
99a2-F	gatcgcacgcgacgtctaatcgctcaaggcgcactcc
99a2-R	cctggtcgagcttaccgagacacgaattatgcagtgatttacgacc
ddh-F	tctcggtaagctcgaccagg
ddh-R	ttagacgtcgcgtgcgatc
UP-A-F	ggcaagctaaatttcggtgc
UP-A-R	attaattaatgggccatcctctgtgcaaacaa
dapA-cat-F	gaggatggcccattaattaatctcgagtgtgac
dapA-cat-R	cttcggttcgatggactattacgccccgccctgcca
dapA-F	tagtccatcgaaccgaagtaaggaacgtaccattgagacacttgtttgcacagag-gatggcccatgttcacgggaagtattgtc
dapA-R	cagttccttacatgcccatt
UP-C-F	gtgatgcggtttttggagtaatc
UP-C-R	attaattaataactacctcgtgtcaggggatc
lysC-cat-F	cgaggtagttattaattaatctcgagtgtgac
lysC-cat-R	cttcggttcgatggactattacgccccgccctgcca
lysC-F	tagtccatcgaaccgaagtaagggtgccaaggctgaaaatggatcccctgacacgaggtag-ttatgtctgaaattgttgtctcca
lysC-R	gaacggttccagtacgcc
EG-F	tcggcgaattgcctcaccaaaaccttccgccgacgg
EG-R	atcccgttaaccatcgccatcaacgcagattcgctatccacat
EGV-F	ctcattaggcaccccaggcctaaggccgcaatccctcgattgctg
EGV-R	tgattggttcttaacatggtttaatatagcttcatgaaccccattcaactggacactttgct

Abbreviations: Ap^R, ampicillin resistance; Km^R, kanamycin resistance.

Table S2. The binding energy of LysG wild-type and lysine.

Name	Binding energy
lys-1	-6.00
lys-2	-5.80
lys-3	-5.66
lys-4	-5.57
lys-5	-4.84
lys-6	-4.62
lys-7	-4.57
lys-8	-4.35
lys-9	-3.78
lys-10	-3.33

Table S3. The binding energy of LysG^M and lysine.

Name	Binding energy
lys-1	-5.73
lys-2	-5.64
lys-3	-5.58
lys-4	-5.55
lys-5	-5.18
lys-6	-4.94
lys-7	-4.60
lys-8	-4.10
lys-9	-3.34
lys-10	-2.45
